# Outcomes of pregnant women exposed to Sotrovimab for the treatment of COVID-19 in the BA.1 Omicron predominant era (PRESTO)

**DOI:** 10.1186/s12879-023-08198-9

**Published:** 2023-04-26

**Authors:** Jessica J. Tuan, Manas Sharma, Jehanzeb Kayani, Matthew W. Davis, Dayna McManus, Jeffrey E. Topal, Onyema Ogbuagu

**Affiliations:** 1 Yale University School of Medicine, 333 Cedar Street, PO Box 208022, New Haven, CT 06510 USA; 2 Yale Department of Medicine, Section of Infectious Diseases, Yale University School of Medicine, 333 Cedar Street, PO Box 208022, New Haven, CT 06510 USA; 3 Yale AIDS Program, Yale University School of Medicine, 135 College Street, Suite 323, New Haven, CT 06510 USA; 4 Department of Pharmacy Services, Yale New Haven Hospital, 20 York Street, New Haven, CT 06510 USA

**Keywords:** Sotrovimab, COVID-19, Omicron, Pregnancy, Monoclonal antibodies

## Abstract

**Background:**

Sotrovimab, a monoclonal antibody with efficacy against SARS-CoV-2 including certain Omicron variants, has been used in treatment of mild-moderate COVID-19. Limited data exists regarding its use in pregnant women.

**Methods:**

Electronic medical record review of pregnant COVID-19 patients treated with sotrovimab from 12/30/21 − 1/31/22 (Yale New Haven Health Hospital System [YNHHS]) was performed. Included were pregnant individuals ≥ 12 years, weighing ≥ 40 kg, with positive SARS-CoV-2 test (within 10 days). Those receiving care outside YNHHS or receiving other SARS-CoV-2 treatment were excluded. We assessed demographics, medical history, and Monoclonal Antibody Screening Score (MASS). The primary composite clinical outcome assessed included emergency department (ED) visit < 24 h, hospitalization, intensive care unit (ICU) admission, and/or death within 29 days of sotrovimab. Secondarily, adverse feto-maternal outcomes and events for neonates were assessed at birth or through the end of the study period, which was 8/15/22.

**Results:**

Among 22 subjects, median age was 32 years and body mass index was 27 kg/m^2^. 63% were Caucasian, 9% Hispanic, 14% African-American, and 9% Asian. 9% had diabetes and sickle cell disease. 5% had well-controlled HIV. 18%, 46%, and 36% received sotrovimab in trimester 1, 2, and 3, respectively. No infusion/allergic reactions occurred. MASS values were < 4. Only 12/22 (55%) received complete primary vaccination (46% mRNA-1273; 46% BNT162b2; 8% JNJ-78,436,735); none received a booster.

**Conclusions:**

Pregnant COVID-19 patients receiving sotrovimab at our center tolerated it well with good clinical outcomes. Pregnancy and neonatal complications did not appear sotrovimab-related. Though a limited sample, our data helps elucidate the safety and tolerability of sotrovimab in pregnant women.

## Background

Sotrovimab, a monoclonal antibody with efficacy against SARS-CoV-2 including certain Omicron variants (BA.1 and BA.1.1), was authorized and used for the treatment of mild to moderate COVID-19. Limited data exists regarding its safety and efficacy in pregnant women [1, 2]. Thus, data is needed on safety and treatment outcomes associated with the use of sotrovimab in pregnancy, a condition that confers a greater risk of severe COVID-19.

## Methods

A review of electronic medical records (EMRs) of all pregnant COVID-19 patients whom received sotrovimab infusions from December 30, 2021 to January 31, 2022 at the Yale New Haven Health Hospital System [YNHHS] was performed. At that time in Connecticut, the SARS-CoV-2 Omicron subvariant BA.1 was predominant at 99.9% and sotrovimab was the only authorized monoclonal antibody treatment recommended for use, as it retained activity against the subvariant.

Inclusion criteria were pregnant individuals (≥ 12 years; ≥40 kg) with positive SARS-CoV-2 test (within 10 days) receiving sotrovimab in ambulatory YNHHS settings. Those receiving care outside YNHHS and recipients of other SARS-CoV-2 treatments were excluded. We assessed subject demographics, medical history (including medical co-morbidities such as diabetes mellitus, chronic lung disease, chronic kidney disease [stage 3 or higher], sickle cell disease, or immunocompromising conditions such as HIV; COVID-19 vaccination status), Monoclonal Antibody Screening Score (MASS) [3], and COVID-19 Pandemic Vulnerability Index (PVI) utilizing the PVI model [4]. The MASS is a score which stratifies patients based on risk of hospitalization. The scoring was based on the original U.S. FDA EUA criteria for increased risk of hospitalization for COVID-19 released in November 2020. A score of 4 or greater represents increased risk of hospitalization (maximum score of 18). The COVID-19 PVI assesses COVID-19 risk based on timeframe and regionality (including county).

The primary composite clinical outcome assessed included all-cause hospitalization (including oxygenation) or emergency department (ED) visit < 24 h, and/or ICU admission, and/or death at 29 days post-sotrovimab infusion. Adverse side effects of infusion or allergic reactions within 1 h of sotrovimab were also captured as reported. Secondarily, adverse feto-maternal outcomes assessed included maternal pregnancy-related (e.g. premature labor defined as labor occurring between weeks 20–37; premature rupture of membranes defined as rupture of the amniotic sac occurring prior to labor onset) and neonatal conditions (if available), neonatal intensive care unit (ICU) admission, neonatal respiratory distress syndrome; birth/neurodevelopmental defects (assessed until August 15, 2022 which was the last estimated date of confinement of subjects in our cohort).

Descriptive statistics were utilized to analyze and report demographic and clinical characteristics as well as frequency of specific predetermined feto-maternal and clinical outcomes among eligible subjects for whom the specific characteristic or outcomes was assessed.

## Results

Among 22 subjects, the median age was 31.5 years (see Table 1). The median body mass index was 27.28 kg/m^2^ (IQR 25.6 to 31.7 kg/m^2^). 63% (14/22) were Caucasian, 13.6% (3/22) African-American, 9.1% (2/22) Asian, and 4.5% (1/22) were of unknown race. 9% reported being of (2/22) Hispanic ethnicity. Regarding comorbidities, 9.1% (2/22) had diabetes and sickle cell disease, respectively. One subject had HIV infection and was virologically suppressed (undetectable HIV viral load, CD4 count 962 cells/µl) on antiretroviral therapy. None had another immunosuppressive condition (e.g. active cancer, solid organ transplant recipient, on immunosuppressants). Of note, one subject had a documented prior history of COVID-19 prior to this study. The COVID-19 unvaccinated subject history had a prior documented history of COVID-19 approximately 4 months prior to the recent COVID-19 diagnosis.

**Table 1:** Demographics and Characteristics of Pregnant Women Exposed to Sotrovimab (n = 22) for COVID-19 Treatment (Omicron Predominant Era)

	n (%)	
Median Age **(years)**		32
Median BMI **(kg/m**^**2**^**)**		27
Race/Ethnicity		
• **Caucasian**	3 (63)	
• **Hispanic**	2 (9)	
• **African-American**	3 (14)	
• **Asian**	2 (9)	
Co-Morbidities		
• **Diabetes Mellitus**	2 (9)	
• **Sickle Cell Disease**	2 (9)	
• **HIV**	1 (5)	
Trimester of sotrovimab receipt		
• **Trimester 1**	4 (18)	
• **Trimester 2**	10 (46)	
• **Trimester 3**	8 (36)	
Adverse Events		
• **Infusion reactions**	0 (0)	
• **Allergic reactions**	0 (0)	
Monoclonal Antibody Screening Score (MASS)		
• **< 4**	22 (100)	
COVID-19 Vaccination		
• Primary series complete	12 (55)	
• **mRNA-1273**	5 (46)	
• **BNT162b2**	5 (46)	
• **JNJ-78,436,735**	1 (8)	
• Booster vaccination complete	0 (0)	

Regarding COVID-19, the median time from symptom-onset to sotrovimab infusion was 5.5 days (IQR 4 to 7 days). Almost half (46%) of participants were in the second trimester of pregnancy at the time of their COVID-19 diagnosis, while 18% were in the first trimester of pregnancy. All subjects had MASS values < 4 [scale 0–18] at time of treatment. Median subject COVID-19 PVI was 0.53. COVID-19 vaccination review revealed that only 12/22 (55%) had received a complete primary series (defined as 2-series for mRNA COVID-19 vaccine or 1-dose for JNJ-78,436,735 COVID-19 vaccine) [45.8% mRNA-1273; 45.8% BNT162b2; 8.3% JNJ-78,436,735]. No subjects had received a COVID-19 booster vaccination. Refer to Table 1 for subject demographics and clinical characteristics.

During the period of the study, no immediate infusion or allergic reactions were reported. Regarding outcomes, within 29 days post-receipt of sotrovimab, there were no maternal COVID-19-related hospitalizations, ICU admissions, or deaths. One subject was hospitalized for post-partum pyelonephritis complicated by *E. coli* bacteremia; another had an ED visit for post-partum vaginal bleeding. None had pre-eclampsia, eclampsia, gestational diabetes, peripartum cardiomyopathy. 9% (2/22) had premature labor and premature rupture of membranes, respectively. Median gestational age at birth was 38.9 weeks. Median infant birth weight was 3220 g. Only 1/22 (4.5%) required neonatal ICU admission due to omphalocele with bowel atresia in the context of fetal growth restriction and had late preterm delivery. Of note, this neonate was prenatally diagnosed with omphalocele prior to receipt of sotrovimab and the neonate’s mother had a personal congenital defect history. There were no abortions, fetal losses, or other birth/neurodevelopmental defects noted. Refer to Table 2 for outcomes of pregnant women and neonates exposed to sotrovimab.

**Table 2:** Outcomes of Pregnant Women and Neonates Exposed to Sotrovimab (n = 22) for COVID-19 Treatment (Omicron Predominant Era)*

	n (%)	
Median Infant Birth Weight (g)		3220
Median Gestational Age at Birth (weeks)		38.9
Premature labor	2 (9)	
Premature rupture of membranes	2 (9)	
Deaths	0 (0)	
Hospitalizations	1 (5)	
• Post-partum pyelonephritis	1 (5)	
ED Visit	1 (5)	
• Post-partum vaginal bleed	1 (5)	
Abortions	0 (0)	
Neurodevelopmental Defects	0 (0)	
Birth Defects	1 (5)	
• Prenatally diagnosed omphalocele (prior to sotrovimab) in mother with congenital defect history	1 (5)	

*There were no maternal ICU admissions nor fetal losses in the cohort

## Conclusion and discussion

In summary, our study demonstrated that pregnant patients diagnosed with COVID-19 who received sotrovimab tolerated it well with good clinical outcomes. The study occurred at a time when rates of SARS-CoV-2 Omicron subvariant BA.1 were 99.9% in Connecticut. These subjects did not experience any COVID-19-related ED visit or hospitalization. Pregnancy and neonatal complications that occurred did not appear related to sotrovimab.

As pregnant women are at increased risk of developing severe disease due to COVID-19, understanding the safety, tolerability and efficacy of treatments for COVID-19, such as monoclonal antibodies, is essential. This information is also important for women of childbearing potential who may become pregnant after receipt of a monoclonal antibody with a long half-life (t_1/2_) (sotrovimab’s t_1/2_ is approximately 45 days), given that fetal exposure may occur when mature placenta forms (typically greater than 14 weeks), as evidence suggests that IgG-based therapies cross the placenta by this time [5].

The sotrovimab efficacy data arises from the COVID-19 Monoclonal Antibody Efficacy Trial–Intent to Care Early (COMET-ICE) Study, a large multi-center randomized clinical trial of 1,057 non-hospitalized adults with mild-moderate COVID-19 receiving sotrovimab compared to placebo (randomized in a 1:1 ratio), which demonstrated a significant reduction in all-cause hospitalization or death 29 days after receipt of sotrovimab (relative to placebo). However, this study excluded pregnant individuals and took place from late 2020–2021, preceding the Omicron era [6, 7]. A multicenter prospective cohort study, the ANRS 0003 S COCOPREV Study, evaluated outcomes of recipients of casirivimab/imdevimab (from September 21, 2021-January 14, 2022) amidst the Delta wave, and outcomes of recipients of sotrovimab (from January 24, 2022-March 3, 2022) amidst the Omicron wave, in a real-world cohort, including immunosuppressed hosts. This study demonstrated that sotrovimab protected those with high risk of COVID-19 progression amidst the Omicron era to a similar extent that casirivimab/imdevimab did amidst the Delta era [8]. However, notably, this cohort does not report on outcomes in pregnant individuals [8].

A study did evaluate and report on outcomes of pregnant women (n = 51) with COVID-19 (deemed high-risk for developing complications related to COVID-19) who received monoclonal antibodies, but this analysis did not include analysis of the monoclonal antibody sotrovimab [9]. In that study, the monoclonal antibodies were well-tolerated and no adverse effects in any mother-fetus pairs were reported [9]. Similarly, among a small cohort of 7 unvaccinated hospitalized pregnant patients who received casirivimab and imdevimab treatment for COVID-19, this monoclonal antibody combination was well-tolerated with no adverse events reported, except for 1 individual who progressed to severe disease [10]. In addition, another study evaluated in a pregnant cohort, the use of monoclonal antibodies (bamlanivimab and etesevimab, casirivimab and imdevimab, or sotrovimab) compared to no monoclonal antibody for treatment of SARS-CoV-2. The study demonstrated that adverse events after monoclonal antibody treatment were mild and rare. In addition, there was no difference in obstetric-related safety outcomes or COVID-19-related outcomes and non-COVID-19 related hospital admissions between the two arms [11]. Notably, neonatal outcomes were not fully described due to a short follow-up period. However, these data, in addition to our study, collectively suggest that COVID-19 monoclonal antibodies are well-tolerated and likely safe in pregnancy, such that benefits of use may outweigh potential risks.

Limitations of our study include limited sample size such that low frequency adverse events could have been undetected. It is important to highlight that as most of our study subjects had completed the first trimester of pregnancy (a period in which the fetus is most vulnerable to developmental complications) by the time of their infusions, this could also have influenced our observed lack of drug-related adverse feto-maternal outcomes. Furthermore, longer-term follow-up for neonatal and infant outcomes longitudinally may be needed to detect neurodevelopmental disorders. In our study, we had a relatively small sample size which may explain the lower event rate of obstetric events (e.g. pre-eclampsia, miscarriage/abortion) observed. Another potential limitation of our study is that our cohort of pregnant women could have been those who had an increased risk for severe COVID-19 versus being non-hospitalized community-dwelling pregnant women. The lack of a control group along with sample size limits the ability to determine efficacy of treatments. That stated, it is remarkable that in spite of the reduced susceptibility of the BA.1 variant to sotrovimab (4-fold less compared to wild-type controls), no COVID-19 hospitalizations or death occurred in an at-risk group. Ultimately, our study adds to the very sparse existing data, helping to elucidate the safety and tolerability of sotrovimab in pregnant women amidst the COVID-19 pandemic in the Omicron era.

## Data Availability

Data from this study are available but protected under the Yale Institutional Review Board given the sensitive nature of patient health information; therefore, restrictions apply to the availability of these data, which were used under license for the current study, and so are not publicly available. Data are however available from the authors upon reasonable request and with permission of Yale. Please contact the corresponding author Dr. Jessica Tuan (e-mail: jessica.tuan@yale.edu) if there is a request for study data.

## Declarations

### Research statement

All methods were carried out in accordance with relevant guidelines and regulations.
